# Evidence for oligodendrocyte progenitor cell heterogeneity in the adult mouse brain

**DOI:** 10.1038/s41598-022-17081-7

**Published:** 2022-07-28

**Authors:** Rebecca M. Beiter, Courtney Rivet-Noor, Andrea R. Merchak, Robin Bai, David M. Johanson, Erica Slogar, Katia Sol-Church, Christopher C. Overall, Alban Gaultier

**Affiliations:** 1 Department of Neuroscience, Center for Brain Immunology and Glia, University of Virginia School of Medicine, Charlottesville, VA 22908 USA; 2 Graduate Program in Neuroscience, University of Virginia School of Medicine, Charlottesville, VA 22908 USA; 3 Genome Analysis and Technology Core, University of Virginia School of Medicine, Charlottesville, VA 22908 USA

**Keywords:** Neuroscience, Glial biology, Oligodendrocyte

## Abstract

Oligodendrocyte progenitor cells (OPCs) account for approximately 5% of the adult brain and have been historically studied for their role in myelination. In the adult brain, OPCs maintain their proliferative capacity and ability to differentiate into oligodendrocytes throughout adulthood, even though relatively few mature oligodendrocytes are produced post-developmental myelination. Recent work has begun to demonstrate that OPCs likely perform multiple functions in both homeostasis and disease and can significantly impact behavioral phenotypes such as food intake and depressive symptoms. However, the exact mechanisms through which OPCs might influence brain function remain unclear. The first step in further exploration of OPC function is to profile the transcriptional repertoire and assess the heterogeneity of adult OPCs. In this work, we demonstrate that adult OPCs are transcriptionally diverse and separate into two distinct populations in the homeostatic brain. These two groups show distinct transcriptional signatures and enrichment of biological processes unique to individual OPC populations. We have validated these OPC populations using multiple methods, including multiplex RNA in situ hybridization and RNA flow cytometry. This study provides an important resource that profiles the transcriptome of adult OPCs and will provide a toolbox for further investigation into novel OPC functions.

## Introduction

First described in the early 1980’s, oligodendrocyte progenitor cells (OPCs) are the fourth major glial subtype present in the brain. Depending on the region examined, OPCs makeup anywhere from 2 to 8% of the cells in the adult central nervous system (CNS)^1,2^. Adult OPCs belong to the same population of progenitors that give rise to oligodendrocytes during CNS development. However, a large fraction of OPCs do not differentiate, but instead remain in a progenitor state throughout adulthood, a property not consistent with the relatively small need to generate new oligodendrocytes^2–6^. While it has been demonstrated that the differentiation of OPCs into myelinating oligodendrocytes is critical for processes such as motor learning during adulthood, recent evidence indicates that mature oligodendrocytes are a relatively stable population in the adult brain^7–9^. The slow rate at which oligodendrocytes are replaced throughout life does not correlate with the maintenance of a highly dynamic and energetically costly population of OPCs^7,8,10^. With this discordance between the dynamics of the OPC population and the relatively small need for newly differentiated oligodendrocytes in adulthood, the field has begun to explore alternate functions of adult OPCs^11,12^.

Under homeostatic conditions, OPCs express distinct ion channel profiles that vary with both the brain region and developmental stage of the organism, indicating that subpopulations of OPCs maintain unique electrical properties and therefore may be performing multiple functions within the brain^13^. Furthermore, loss of OPCs, either globally or regionally, has been shown to result in significant depressive-like behavior, persistent weight-gain and leptin-insensitivity, as well as microglial activation and subsequent neuronal death^14–16^. In pathological conditions, OPCs can upregulate cytokine production in response to IL-17 signaling and greatly contribute to CNS pathogenesis^17^. Surprisingly, OPCs also upregulate antigen presentation machinery in the demyelinating CNS and can regulate T cell function^18–20^. Taken together, these studies illustrate the dynamic role OPCs can play in the adult CNS and build a strong case in support of exploring adult OPC diversity at the transcriptional level. A profiling of the overall transcriptional profiles of adult OPCs will provide an important resource for further functional investigation of OPCs in the CNS.

Here, we have developed an inducible OPC reporter mouse strain, which expresses YFP in PDGFRα-expressing cells after tamoxifen administration. After validation of the model, we used this tool to isolate OPCs from the adult brain by fluorescent activated cell sorting (FACS) and perform single-cell sequencing. We demonstrate the presence of two novel populations of transcriptionally distinct OPCs in the adult brain. Gene Ontology (GO) term analysis and gene expression analysis of identified OPC subtypes support specialization of OPCs, encompassing potential functions such as extra-cellular matrix modulation and neuronal regulation. Sequencing results were validated in vivo by staining for cluster-specific genes identified from our sequencing dataset using RNAscope and confirming that these two clusters do not overlap using RNA flow cytometry. Taken together, our results present a unique toolbox to support functional exploration of OPCs under homeostatic and pathological conditions.

## Methods

### Animals

PDGFRα-Cre^ER^ mice (Jackson #018280) were crossed to R26-EYFP (Jackson, #006148) animals to generate PDGFRα-Cre^ER^; R26-EYFP mice, a previously described model^21^. C57B/6J used as adults were purchased from Jackson. Mice were maintained on a 12-h light/dark cycle with lights on at 7am. Behavior was performed on mice used in single-cell sequencing run 1. Testing consisted of sucrose preference, elevated plus maze, open field, and forced swim test. All animal experiments were approved and complied with regulations of the Institutional Animal Care and Use Committee at the University of Virginia (protocol #3918). All experiments were conducted and reported according to ARRIVE guidelines (https://arriveguidelines.org/arrive-guidelines).

### Tamoxifen injections

Tamoxifen (C8267, Sigma-Aldrich) was dissolved in corn oil at 37 °C overnight at 20 mg/mL. Tamoxifen was administered *i.p*. at 200 mg/kg with a maximum dose of 4 mg per injection. For single-cell sequencing experiments, six-week-old mice were given two injections of tamoxifen, 3 days apart. For validation of Cre recombination in PDGFRα-Cre^ER^; R26-EYFP brains, 5- to 6-week-old mice were injected with 0, 1, 2, or 3 doses of tamoxifen, each given 3 days apart. For those mice receiving three doses of tamoxifen, the final dose was given at 150 mg/kg.

### Immunofluorescence

Mice were deeply anesthetized with pentobarbital and subsequently perfused with 5 units/mL heparin in saline followed by 10% buffered formalin, each for approximately one minute. Brains were rapidly dissected and post-fixed in 10% buffered formalin overnight at 4 °C. Tissue was then transferred into 30% sucrose in PBS and allowed to sink for at least 24 h. Brains were frozen in OCT, sectioned, and stored in PBS plus 0.02% NaAz until further staining.

Tissue was blocked with PBS, 1% BSA, 0.5% Triton-X 100, 2% normal donkey serum, and 1:200 CD16/CD32 (14-0161-82, 1:200, eBioscience) for at least 1 h at room temperature. For stains utilizing a mouse primary antibody, tissue was blocked in Mouse-on-Mouse Blocking Reagent (MKB-2213, Vector Laboratories) according to manufacturer’s instructions for at least 1 h at room temperature. Tissue was incubated in primary antibodies overnight at 4 °C with gentle agitation. Tissue was washed three times in TBS containing 0.3% Triton-X 100 and incubated in secondary antibodies overnight at 4 °C with gentle agitation. Following secondary incubation, tissue was stained with Hoechst (1:700, ThermoFisher Scientific, H3570) for 10 min at room temperature, washed three times in TBS containing 0.3% Triton-X 100, and mounted on slides using Aqua Mount Slide Mounting Media (Lerner Laboratories). Images were collected on a Leica TCS SP8 confocal microscope and processed using Fiji.

### Antibodies for immunofluorescence

Primary antibodies used for immunofluorescence were PDGFRα (1:200, R&D Systems, AF1062), Olig2 (1:200, Millipore, MABN50), GFP-488 (1:400, Fisher Scientific, A21311), and GFP (1:1000, Invitrogen, A10262). Secondary antibodies used were Donkey anti-Goat Cy3 (2 μg/mL, Jackson ImmunoResearch, 705-165-147), Donkey anti-Mouse 647 (2 μg/mL, Jackson ImmunoResearch, 715-605-150), Donkey anti-Mouse 546 (2 μg/mL, Life Technologies, A10036), Donkey anti-Chicken 488 (2 μg/mL, Jackson ImmunoResearch, 703-545-155), and Donkey anti-Goat 647 (2 μg/mL, Invitrogen, A21447).

### Isolation of CNS cells

To prepare cells for single-cell sequencing, adult mice (8–20 weeks) were anesthetized with pentobarbital and subsequently perfused with 5 units/mL heparin in saline for approximately 1 min. Brains were rapidly dissected and finely minced. For single-cell sequencing experiments, tissue was digested in HBSS with calcium and magnesium (Gibco, 14025-092) supplemented with 20 units per mL papain (Worthington Biochemical LS003126) and 50 units per mL DNase (Worthington Biochemical, LS002139). Tissue was digested at 37 °C with gentle shaking for 45 min, with trituration after every 15-min interval to dissociate the tissue. Following digestion, a 40% Percoll gradient (GE Healthcare, 17-0891-01) was used to remove myelin and other debris from the samples. Resulting single-cell suspensions from 4 to 5 mice were pooled for each sequencing sample and subsequently stained for FACS sorting.

### FACS sorting

For single-cell sequencing experiments, single-cell suspensions were stained for 30 min at room temperature with the following antibodies: O4-APC (O4, 10 µL/test, Miltenyi, 130-095-891), CD11b-e450 (M1/70, 0.5 µL/test, eBioscience, 48-0112-82), TER119-APC/Cy7 (TER-119, 1.25 µL/test, Biolegend, 116223), PDGFRα-PE/Cy7 (APA5, 0.625 µL/test, Invitrogen, 25-1401-82), CD45-PerCP/Cy5.5 (30-F11, 0.5 µL/test, eBioscience, 45-0451-82), and CD16/31 (93, 0.5 µL/test, Invitrogen, 14-0161-82). Viability was determined using Ghost Dye Violet 510 (0.5 µL/test, Tonbo biosciences, 13-0870). Cells were sorted using a 16-color BD influx cell sorter. Cells used for sequencing were gated on live/singlets/TER119−/CD45−/CD11b−/YFP+. Following sorting, cells were washed three times with 0.04% BSA and then processed for sequencing according to the 10× Genomics protocol.

### Single-cell sequencing and analysis

#### Library preparation and sequencing

Samples were processed for single-cell sequencing according to manufacturer’s instructions using the Chromium Next GEM Single-cell 3ʹ Reagent Kit (10× Genomics) and Chromium Controller (10× Genomics). Single-cell libraries were sequenced using the NextSeq 500 Sequencing System (Illumina). Library preparation and sequencing was completed by the Genome Analysis and Technology Core at the University of Virginia.

#### Quantification

All steps of the quantification process were performed with Cellranger. The fastq files for the samples were quantified using the mkfastq utility and were quantified against the mm10 mouse genome with the count utility.

#### Pre-processing

Seurat (version 3, https://satijalab.org/seurat/) was used for the single-cell analysis^22,23^, and for each of the sequencing run datasets, we followed the same procedure. First, a QC step was performed to identify and remove cells that were potential outliers. This included removing potential multiplets (i.e., cells that had clear outlier gene expression) and cells that had approximately ten percent or more of mitochondrial gene expression (i.e., cells that were likely to have high technical variation). After filtering out these suspect cells, the data was normalized and log-transformed (using the ‘LogNormalize’ method), unwanted sources of technical variation were regressed out (i.e., the number of detected molecules and mitochondrial contribution to gene expression)^24^, and the counts were scaled.

#### Integration

To make comparative analyses possible between multiple sequencing run datasets, the datasets were integrated with Seurat using the alignment strategy described previously^22^. The first step was to select the genes to be used as the basis for the alignment. The union of the 1000 genes with highest variability in each of the datasets was taken and then filtered this down to only those genes found in each of the datasets, resulting in 2285 genes for the alignment. Next, the common sources of variation between the six datasets (3 sequencing runs with 2 samples each) was identified by running a canonical correlation analysis (CCA) with the highly variable genes as features. By examining the strength of the thirty calculated canonical correlations (CCs), that the first twelve CCs were identified to be driving the variation between the datasets. The subspaces^22^ (i.e., the first twelve CCs) were then aligned, resulting in an integrated dataset with features on a common scale.

#### Analysis

Seurat was used on the aligned dataset to identify eight clusters of cells, and then used t-SNE to visualize the similarity between cells. Next, cell types were assigned to these clusters based upon the expression of pre-defined marker genes, and then identified cluster markers by finding the differentially expressed genes in one cluster compared to all other clusters (one-vs-all). Using the gene markers for each cluster, gene set analysis (Fisher’s exact test, as implemented in the clusterProfiler Bioconductor package^25^) was subsequently used to identify GO gene sets that were enriched. To better identify markers that differentiated the two OPC clusters and the cluster of VLMCs from each other, the other five clusters were excluded and each of the remaining 3 clusters were compared to the other two individually (one-vs-all) and the differentially expressed genes were identified. As before, these marker genes were used to identify GO gene sets that were functionally enriched.

### Subclustering

Reclustering was performed in Seurat. Briefly, cells from each original cluster of interest were used to create new Seurat objects consisting of only cells from the clusters of interest. Next, the count data was split by sex and re-normalized with the command: “NormalizeData(object, verbose = FALSE, normalization.method = “LogNormalize”, scale.factor = 10,000, assay = “RNA”)”. The Seurat function “FindVariableFeatures” with the “vst” method was used to identify the 2000 most variable genes in each sex. Next, FindIntegrationAnchors was applied with dims = 1:30 to integrate the data with CCA using the first thirty CC vectors. “IntegrateData” was called to integrate the two sexes. Next, the call: “ScaleData(object, scale.max = 10, model.use = “linear”, use.umi = FALSE, do.scale = TRUE, do.center = TRUE, block.size = 1000, min.cells.to.block = 3000, verbose = FALSE)” was used to re-center and scale the counts matrix for each new Seurat object representing a cluster. “RunPCA(object, npcs = 30, verbose = FALSE)” was used to perform PCA, and “RunTSNE(object,dims.use = 1:14, max_iter = 2000)”, “FindNeighbors(object, reduction = “pca”, dims = 1:14)”, and “FindClusters(object, resolution = 0.6)” were called to identify new sub-clusters. Visualizations were performed using the Seurat function “VlnPlot”.

### RNAscope

C57B/6J mice (8–10 weeks) from Jackson were anesthetized with pentobarbital and subsequently perfused with ice-cold 5 units/mL heparin in saline for approximately 1 min. Brains were rapidly dissected, flash frozen in OCT (Fisher Healthcare, 4585), and stored at − 80 °C until further processing. Frozen tissue was cut sagittally (15 μm), immediately slide-mounted, allowed to dry for approximately 1 h at − 20 °C and then stored at − 80 °C. All tissue was used within 3 months of dissection.

Tissue was processed using the RNAscope Fluorescent Multiplex Reagent Kit (Advanced Cell Diagnostics, 320850) according to manufacturer’s instructions. Briefly, tissue was fixed for 15 min in 10% buffered formalin (Fisher Scientific, 23-245685) at 4 °C, dehydrated, and then incubated in Protease IV (Advanced Cell Diagnostics, 320850) at room temperature for 30 min. Target probes were hybridized to the tissue for 2 h at 40 °C, followed by hybridization of AMP1-FL (30 min, 40 °C), AMP2-FL (15 min, 40 °C), AMP3-FL (30 min, 40 °C), and AMP4-FL (15 min, 40 °C). Samples were counterstained with supplied DAPI or Hoechst 33342 (1:700, ThermoFisher Scientific, H3570) and mounted on slides using ProLong Glass Antifade Mountant (ThermoFisher, P36980). The following target probes were used: *Olig1* (Advanced Cell Diagnostics, 480651-C2), *Olig2* (Advanced Cell Diagnostics, 447091-C2), *Pdgfrα* (Advanced Cell Diagnostics, 480661), *Clusterin* (Advanced Cell Diagnostics, 427891-C3), *Gpr17* (Advanced Cell Diagnostics, 318131-C3), *Cspg4* (Advanced Cell Diagnostics, 404131), *Lumican* (Advanced Cell Diagnostics, 480361-C3), RNAscope 3-plex Positive Control Probes (Advanced Cell Diagnostics, 320881), and RNAscope 3-plex Negative Control Probes (Advanced Cell Diagnostics, 320871). Sections were imaged using a Leica TCS SP8 confocal microscope.

### RNAscope quantification

Following imaging, max projected confocal images were analyzed using CellProfiler Software 4.2.1 (https://cellprofiler.org/). RNA expression per cell was quantified using a modified version of a previously published pipeline^26^. Briefly, automated steps were used to draw nuclear masks and subsequently quantify the number of RNA puncta from each channel that colocalized with each nuclear mask. Threshold values for each channel were set based on negative control images. Automatic nuclear identification was reviewed and any nuclear mask that clearly contained a large group of nuclei or was located on the edge of an image such that part of the nuclei was not visible was excluded from further analysis. Cells were considered positive for an OPC marker (*Pdgfra*, *Olig1*, or *Olig2*) if four or more puncta colocalized with a particular nucleus to account for background in the assay^27^. OPCs were defined by the co-expression of two canonical OPC transcripts encoding for cell surface markers (*Pdgfra* or *Cspg4*) and oligolineage transcription factors (*Olig1* or *Olig2*). The number of transcripts of cluster markers clusterin or Gpr17 were recorded for each identified OPC. OPCs were considered *Clu*− or *GPR17*− if they contained 10 or fewer puncta. For analysis of cells expressing *Lumican*, cells were categorized as also expressing *Pdgfra*, *Olig1*, both, or neither.

### RNA flow cytometry

Isolated cells were stained according to the PrimeFlow RNA Assay kit (ThermoFisher Scientific, 88-18005-204). Probes used include *Olig2* (Affymetrix, VPFVKKV-210), *Gpr17* (Affymetrix, VPGZE6T-210), *Clu* (Affymetrix, VB10-3283998-210) and *Pdgfra* (Affymetrix, VB6-3197712-210). A probe targeting *Actb* (Affymetrix, VB1-10350-210) was used as a positive control to ensure good RNA quality. Samples were run using a 16-color Life Technologies Attune Nxt flow cytometer and data was analyzed using FlowJo software 10.8.1 (https://www.flowjo.com).

### Image acquisition and processing

All images were collected using a Leica TCS SP8 confocal microscope. All image processing was done using Fiji. RNAscope quantification was done using CellProfiler.

### Statistical analysis

Statistical analysis of all data (except single-cell sequencing data) was done using GraphPad Prism 9 (https://www.graphpad.com/scientific-software/prism/). Significance was set at p < 0.05. Figure 7B was analyzed using a one-way repeated measures ANOVA with a Tukey’s post-hoc test. Figure 5B was analyzed using a two-way ANOVA with a Tukey’s post-hoc test.

## Results

### Validation of inducible OPC reporter mouse line

**Figure 1:**
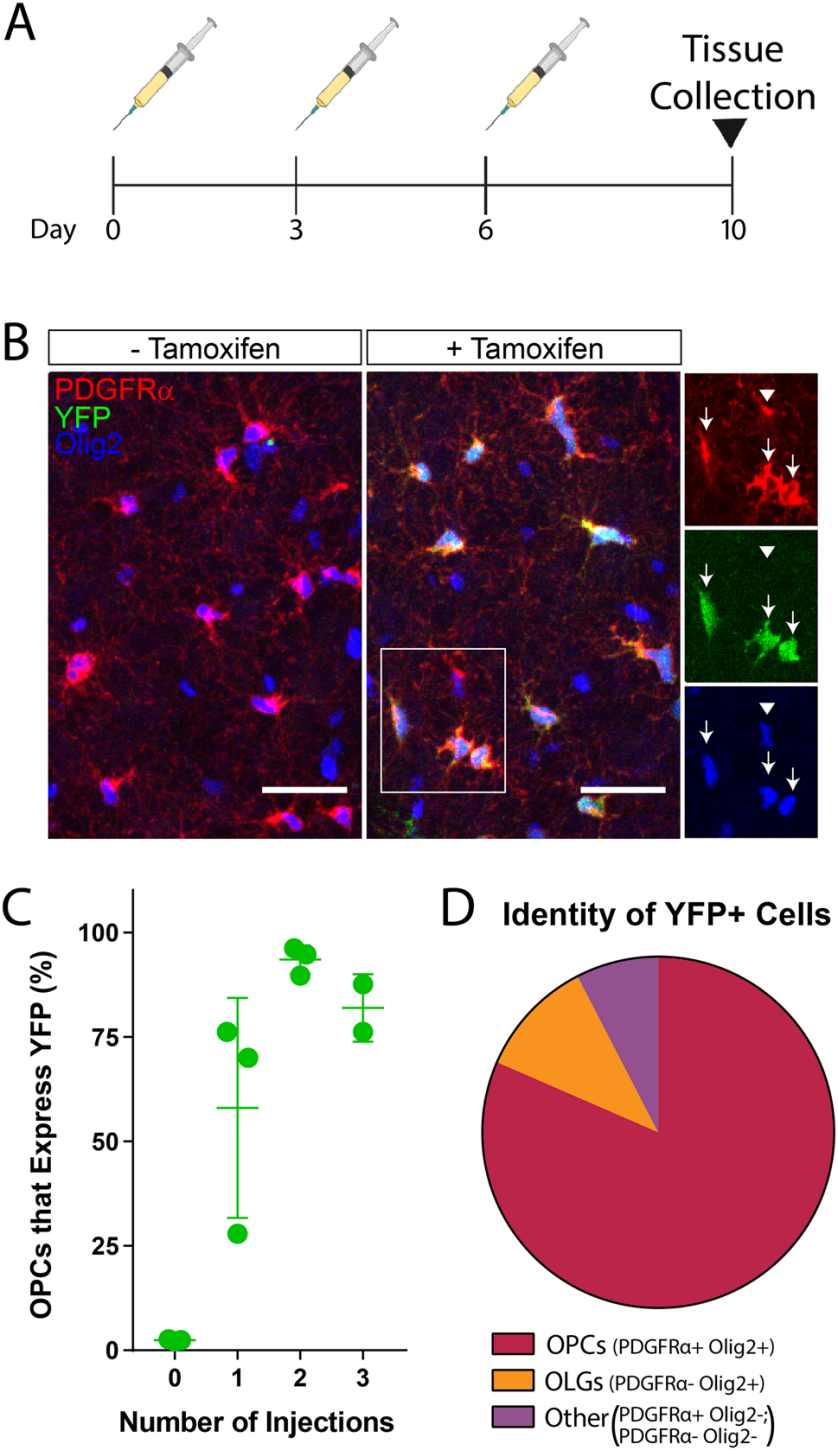
Validation of PDGFRα-Cre^ER^; R26-EYFP reporter mouse. (**A**) Timeline of tamoxifen injections and tissue harvest used to validate YFP expression and titrate optimal tamoxifen dosing paradigm. (**B**) Immunofluorescence of PDGFRα (red), Olig2 (blue), and YFP (green) in PDGFRα-Cre^ER^; R26-eYFP mice receiving no tamoxifen injections (− Tamoxifen) or 2 tamoxifen injections (+ Tamoxifen). Arrows represent OPCs expressing YFP and arrowheads represent OPCs lacking YFP expression. Scale bar = 30 µm. (**C**) Percentage of OPCs (PDGFRα+/Olig2+) that also express YFP following 0, 1, 2, or 3 tamoxifen injections. N = 3 independent experiments, n = 2–3 per group. Error bars represent SEM. (**D**) Proportion of YFP+ cells identified as OPCs (PDGFRα+/Olig2+), Oligodendrocytes (OLG, PDGFRα−/Olig2+), or neither of these cell types (Other, PDGFRα+/Olig2− or PDGFRα−/Olig2−) following 2 tamoxifen injections. N = 2 independent experiments, n = 3 samples. Data quantified in (**C,D**) include images from the prefrontal cortex, hippocampus, and corpus callosum.

To label oligodendrocyte progenitor cells (OPCs) in the adult mouse brain with as little off-target labeling as possible, we utilized a PDGFRα-Cre^ER^; R26-EYFP mouse line^6^. Animals were injected with tamoxifen at 6 weeks of age to avoid labeling the pool of OPCs that differentiate into oligodendrocytes during developmental myelination^28^. Two tamoxifen injections were sufficient to label the majority (93.56% ± 1.98) of OPCs in the brain (Fig. 1A,C). Immunohistochemistry revealed that yellow fluorescent protein positive cells (YFP+) following two tamoxifen injections were composed of OPCs (81.5%; PDGFRα+/Olig2+), mature oligodendrocytes (10.9%, PDGFRα−/Olig2+) or other cell types not belonging to the canonical oligolineage (7.6%, PDGFRα−/Olig2− or PDGFRα+/Olig2−), presumably endothelial cells, as they are also known to express PDGFRα (Fig. 1B,D)^29^. One cohort of mice underwent behavioral testing prior to sequencing experiments to ensure no behavioral phenotypes were observed in this mouse model.

### Isolation and sequencing of YFP+ cells

**Figure 2:**
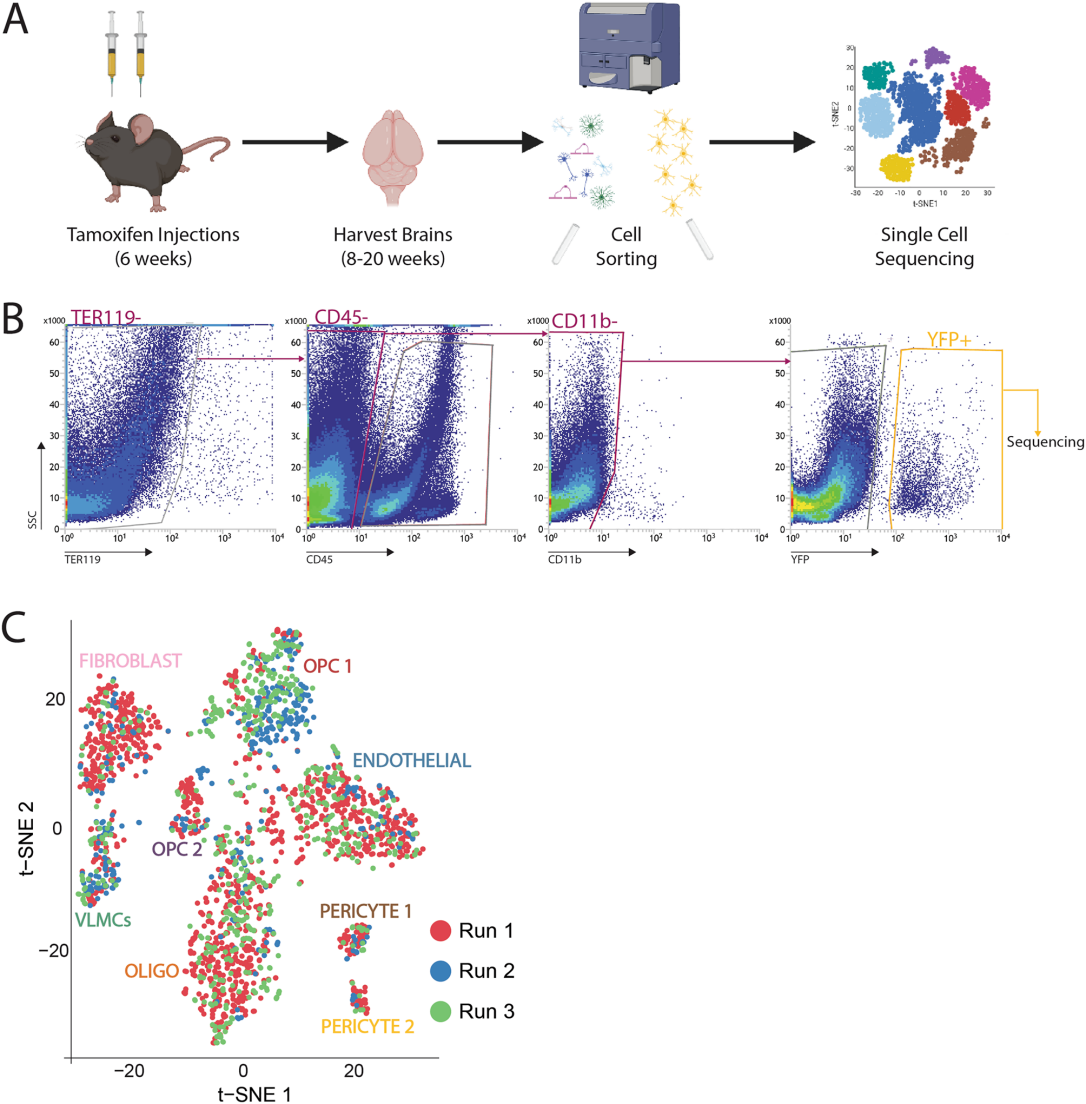
Isolation of YFP+ cells from PDGFRα-Cre^ER^ × YFP brains for sequencing. (**A**) Experimental strategy used for the isolation and single-cell sequencing of cells. (**B**) Gating strategy for YFP+ cell sorting following live/scatter/singlet gating. (**C**) tSNE map depicting cell clusters colored by sequencing run.

Whole brains, including the cerebellum but excluding the spinal cord, were collected from adult PDGFRα-Cre^ER^; R26-EYFP mice and processed into a single-cell suspension for FACS. Four to five brains were pooled for each sample, and male and female brains were processed separately to allow for analysis of potential sex differences in YFP+ cells (Fig. 2A). YFP+ cells were selected by gating on live cells while excluding immune (CD45+) and red blood cells (TER119+), thus ensuring that the population of YFP+ cells collected were viable and highly enriched (Fig. 2B). YFP+ cells were barcoded and prepared for single-cell sequencing using Next GEM reagents and Chromium microfluidics supplied by 10× Genomics. Cell sorting and sequencing was performed three independent times for a total of 6 independently sequenced samples. Unbiased clustering of each independent run revealed overlap between distinct sequencing runs and no clustering of cells driven by sequencing run alone (Fig. 2C). For all future analysis, all sequencing runs were combined to form one dataset, with all sequencing data including cells from both male and female mice.

### Profiling the molecular signature of OPCs in the adult brain

**Figure 3:**
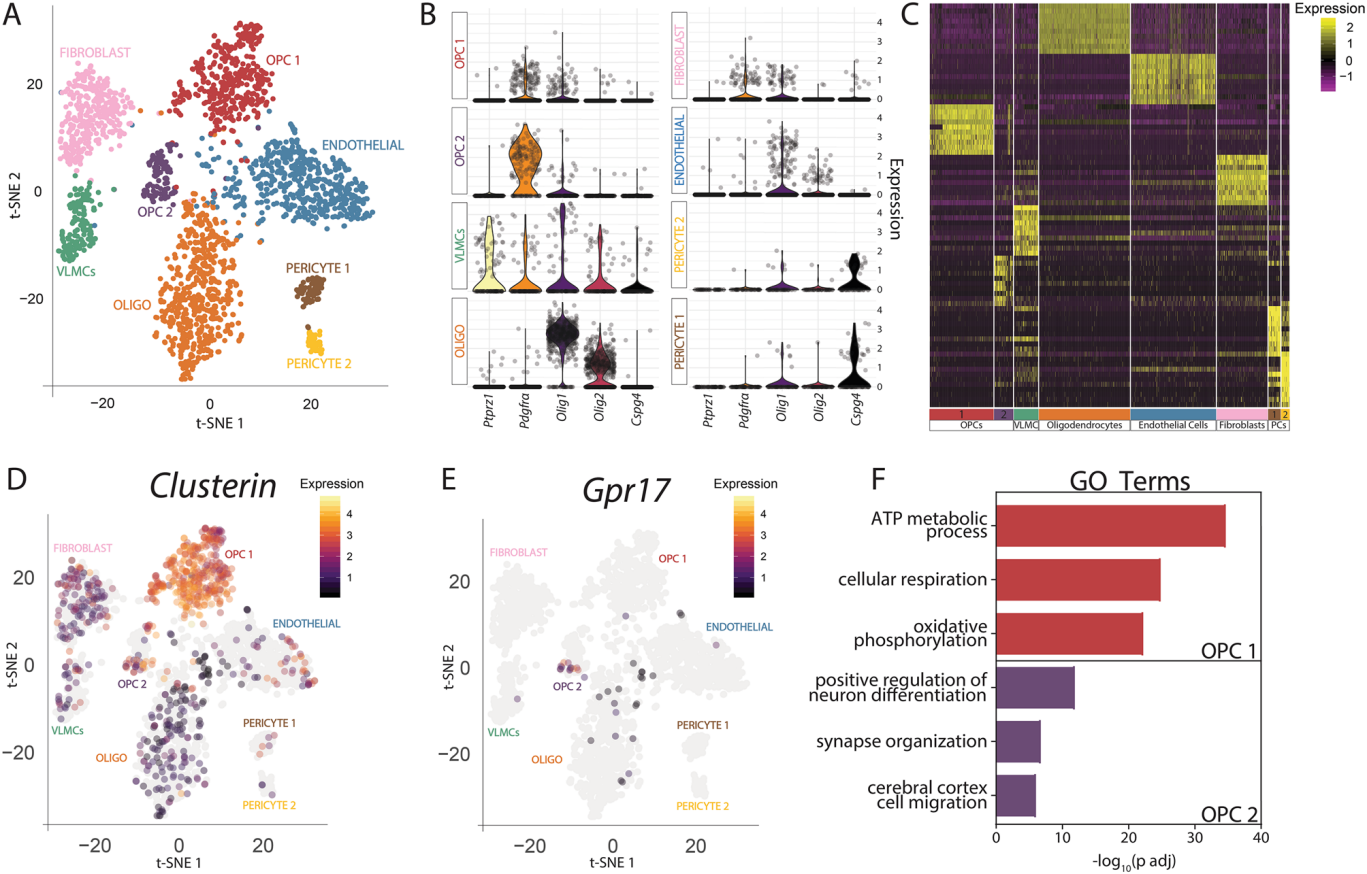
Adult OPCs cluster in two distinct subpopulations. (**A**) tSNE plot of all sequenced cells isolated from PDGFRα-Cre^ER^ reporter brains. Clusters were labeled with cell-type classifications based on expression of common cell-type markers. N = 3 independent experiments, n = 6 biological samples (3 males and 3 females). (**B**) Violin plots depicting expression of common OPC markers in each cluster. Each dot represents a cell. Expression value is plotted on the y-axis. (**C**) Heatmap depicting the scaled and log-normalized expression values of the top 10 most highly enriched genes in each cluster. (**D**) Cell-specific expression of *Clusterin* used for cluster validation of OPC1 overlaid on the tSNE map. (**E**) Cell-specific expression of *Gpr17* used for cluster validation of OPC2 overlaid on the tSNE map. (**F**) Significant GO terms uniquely upregulated in one OPC cluster compared to each OPC cluster and VLMCs. *PCs* pericytes.

Unbiased clustering of sequenced cells using the Seurat package^22,23^ revealed that cells sorted from PDGFRα-Cre^ER^; R26-EYFP brains clustered into 8 distinct populations (Fig. 3A). Mature oligodendrocytes comprised one cluster, having potentially differentiated following initial tamoxifen labeling of PDGFRα expressing progenitor cells. Also captured in the sequencing were four cell types outside the oligolineage that are known to either express PDGFRα or come from PDGFRα-expressing precursors, including one population of fibroblasts, one population of endothelial cells, one population of vascular and leptomeningeal cells (VLMCs), and two populations of pericytes^29–33^. These clusters were identified by expression of known cell type markers such as *Igfbp6* and *Fn1* (fibroblasts), *Tek*, *Pecam1*, and *Kdr* (endothelial cells), *Lum*, *Col1a2*, and *Col3a1* (VLMCs), as well as *Rgs5*, *Pdgfrb*, and *Des* (pericytes) (Supplemental Table 1)^30,34–37^. The remaining 2 clusters of cells (OPC1 and OPC2) expressed at least 2 of the 5 canonical OPC markers *Ptprz*, *PDGFRα*, *Olig1*, *Olig2*, and *Cspg4* (Fig. 3B)^18,38–41^. Importantly, each OPC population expressed a unique transcriptional signature distinct from the gene expression in every other cluster (Fig. 3C). *Olig1* and *Olig2* expression was only detected in a fraction of our OPC clusters, paralleling what has been published by Marques et al.^30^. While single-cell sequencing provides gene expression data on an individual cell basis, its relatively shallow depth of sequencing can result in limited detection of genes with low expression, such as transcription factors^42,43^. This may explain the modest expression of *Olig1* and *Olig2* within our OPC populations. We noted that *Olig1* and *Olig2* were also detected in other populations (endothelial cells and fibroblasts). Future studies will be necessary to test if this signal emanated from contaminating transcripts release by dying oligolineage cells generated by sample preparation or represented real expression. Regardless, further in vivo validation of these clusters, such as done here, is warranted to thoroughly validate the range of transcriptional heterogeneity of OPCs and oligolineage cells.

To further investigate how these clusters of OPCs are distinct from one another, we identified a significantly upregulated gene from each cluster that offered potential indications of distinct functions of these subpopulations. OPC1 expressed high levels of *Clusterin*, a multifunctional protein that has been identified as a risk factor for late-onset Alzheimer Disease (Fig. 3D)^44^. OPC2 cells were positive for G-protein coupled receptor *Gpr17*, the only previously described marker of molecular diversity in OPCs, when compared to all other cell types sequenced (Fig. 3E)^45,46^.

Lastly, to gain insight into how these subpopulations of OPCs potentially differ at the functional level, we analyzed GO terms that were unique to each OPC cluster (Fig. 3F). OPC1 showed upregulation in genes involved in ATP metabolic processes, cellular respiration, and oxidative phosphorylation, including *Chchd10*, *Mdh1*, and *Uqcrq*. OPC2 was characterized by the upregulation in genes involved in the positive regulation of neuron differentiation, synapse organization, and cerebral cortex cell migration, including *Stmn2*, *Pfn2*, and *Dcx* (Supplemental Table 2). Searchable gene expression data for these clusters is available on our website (http://165.22.7.10:3838/seurat_viewer/seurat_viewer_4.Rmd). In sum, our single sequencing data reveals two unique subpopulations of OPCs that reside in the adult brain under homeostatic conditions and support functional diversity based on unique gene expression profile.

### Subclustering further elucidates potential functions of OPC clusters

**Figure 4:**
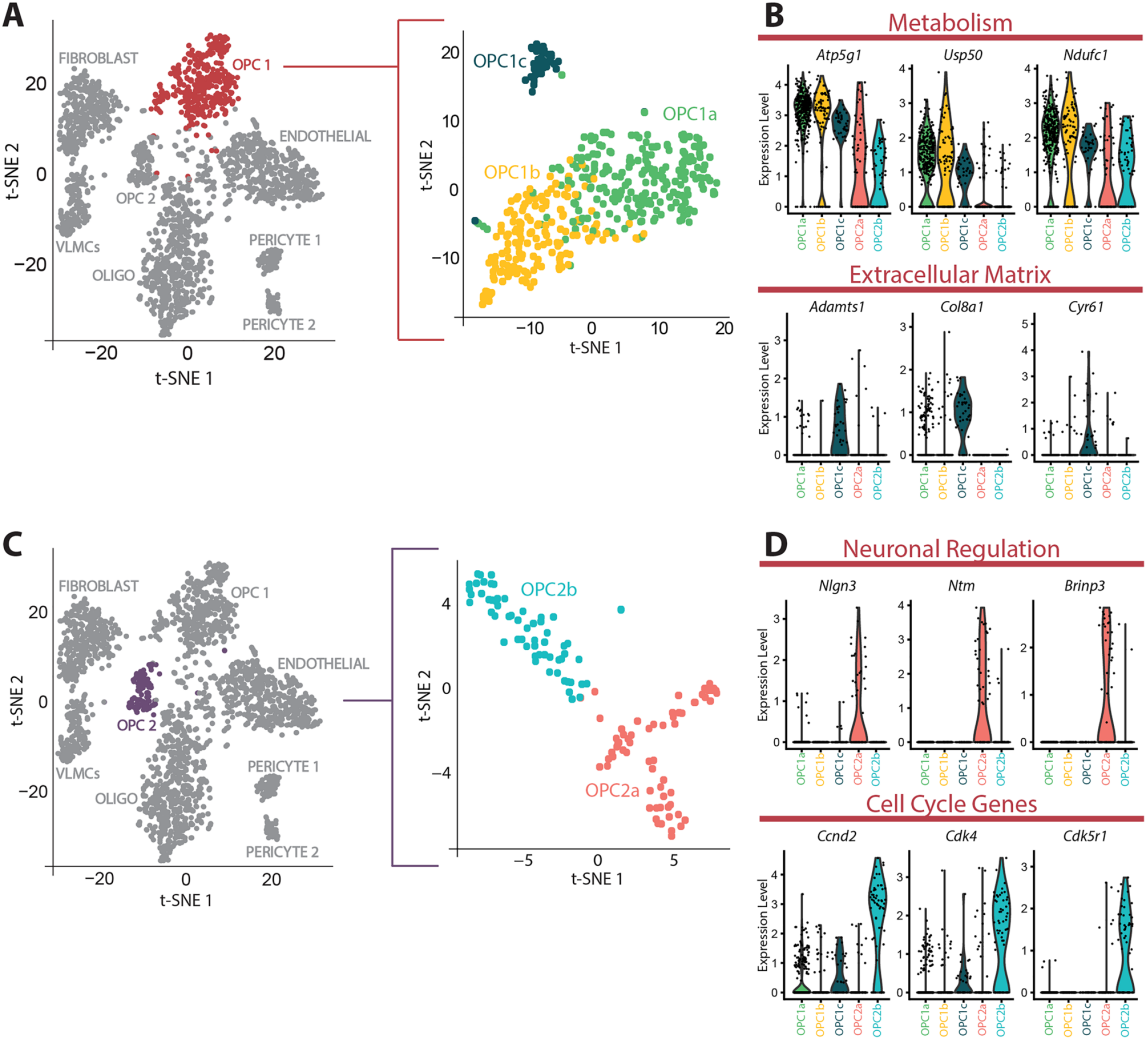
Subclustering reveals potential functions of OPCs. (**A**) Subclustering of OPC1 into 3 subclusters (OPC1a, OPC1b, and OPC1c). (**B**) Violin plots depicting expression of genes related to metabolism that are significantly upregulated in OPC1a and genes related to the extracellular matrix that are significantly upregulated in OPC1c. Each dot represents a cell. (**C**) Subclustering of OPC2 into 2 subclusters (OPC2a and OPC2b). (**D**) Violin plots depicting expression of genes related to neuronal regulation that are significantly upregulated in OPC2a and genes related to the cell cycle that are significantly upregulated in OPC2b. Each dot represents a cell.

Since the inclusion of multiple cell types in a single-cell sequencing dataset has the potential to mask transcriptional differences between subsets of one cell type, we decided to reanalyze each cluster of OPCs individually to bypass this problem. This additional analysis identified 3 subclusters in OPC1 (OPC1a, OPC1b, OPC1c; Fig. 4A). However, OPC1a and OPC1b were very transcriptionally similar, and we believe that together they represent one functional subcluster within OPC1. This analysis also identified 2 subclusters in OPC2 (OPC2a, OPC2b; Fig. 4C). Analysis of genes significantly upregulated in each cluster further highlighted potential alternative functions for each subset of OPCs. For example, OPC1a and OPC1b highly express genes related to metabolic activity (*Atp5g1, Usp50, Ndufc1*; Fig. 4B) and OPC1c shows significant upregulation of genes related to the regulation of the extracellular matrix (*Adamts1*, *Col8a1*, *Cyr61*; Fig. 4B, Supplemental Table 3). Similarly, OPC2a expresses genes related to neuronal regulation (*Nlgn3*, *Ntm*, *Brinp3*; Fig. 2D) and OPC2b expresses genes that are known to regulate the cell cycle (*Cdk4*, *Cdk5r1*, *Ccnd2*; Fig. 4D) and are likely the actively proliferating subset of OPCs that have been previously described (Supplemental Table 3)^33^. Further subsetting our clusters of OPCs into transcriptionally distinct subclusters could lay the foundation for a more targeted exploration of novel functions of OPCs.

### Sex differences in OPCs

**Figure 5:**
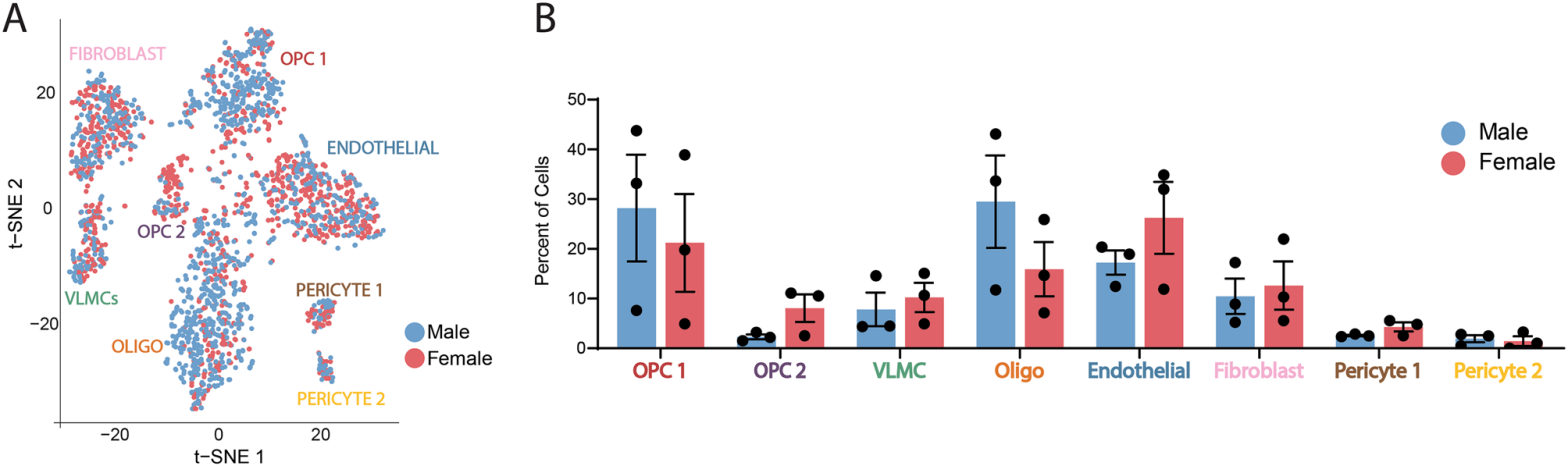
Distribution of PDGFRα positive cells in each cluster based on sex. (**A**) tSNE map depicting cell clusters colored by sex. (**B**) Percent of male and female cells that fall within each cluster. Each dot represents an individual sequencing sample.

Since sex differences have been reported in multiple types of glia including microglia and astrocytes, we investigated whether OPCs isolated from male and female mice were different^47,48^. Cells from male and female animals were present in all 8 clusters (Fig. 5A). We found that there were no significant differences in the distribution of OPC subtypes present in each sex (Fig. 5B). Further investigation into potential sex differences in each cluster showed that the number of significantly different genes between male and female cells in each cluster fell within the range of statistical noise (data not shown). While this data indicates no sex specific OPC signature in the adult brain on a broad transcriptional level, this does not rule out more subtle differences in gene expression or cellular function and further investigation into these potential sex differences is warranted.

### In vivo validation of OPC subpopulations

**Figure 6:**
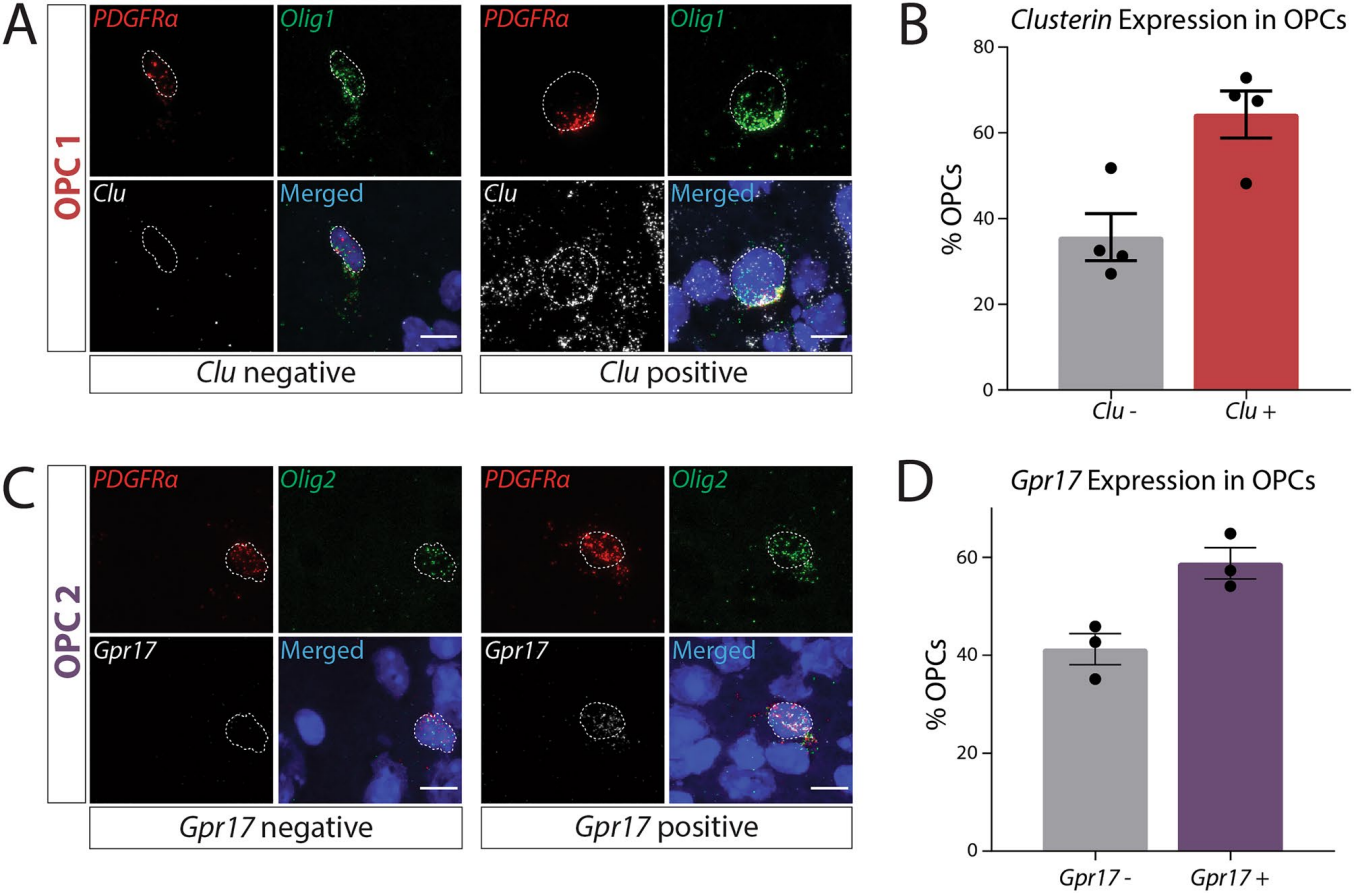
In vivo expression of cluster markers *Clusterin* and *Gpr17* in OPCs. (**A**) RNAscope expression of *Pdgfrα* (red), *Olig1* (green), *Clu* (white), and Hoechst (blue). White dotted line indicates the nuclear outline. (**B**) Quantification of *Clu*+ and *Clu*− OPCs (depicted in (**A**); N = 2 independent experiments, n = 4 individual mice (2 males and 2 females) with 206 total cells analyzed). Each sample includes quantification of OPCs from the cortex, hippocampus, corpus callosum, and cerebellum. (**C**) RNAscope expression of *Pdgfrα* (red), *Olig2* (green), *Gpr17* (white), and Hoechst (blue). White dotted line indicates the nuclear outline. (**D**) Quantification of *Gpr17*+ and Gpr17− OPCs (depicted in (**C**); N = 2 independent experiments, n = 3 individual mice (1 male and 2 females) with 204 total cells analyzed). Each sample includes quantification of OPCs from the cortex, hippocampus, corpus callosum, and cerebellum. In all RNAscope experiments, OPCs were identified as cells co-expressing an OPC surface marker (*Pdgfrα* or *Cspg4*) and an oligolineage transcription factor (*Olig1* or *Olig2*). Scale bar = 10 µm.

Since gene expression can be altered by tissue processing before sequencing, we validated the expression of each OPC cluster marker using RNAscope in the adult mouse brain^49^. OPCs were defined by the co-expression of two canonical OPC transcripts encoding for cell surface markers (*Pdgfra* or *Cspg4*) and oligolineage transcription factors (*Olig1* or *Olig2*). Using a Cell Profiler pipeline to unbiasedly quantify RNA puncta expression per cell^26^, we subsequently quantified expression of each OPC cluster gene. We confirmed that a subset of OPCs (64.32% ± 5.49%) expressed *clusterin*, the OPC1 marker, in vivo, and these cells were present both in the white matter and the gray matter (Fig. 6A,B). Similarly, we used *Gpr17* as a marker for the remaining subset of OPCs (OPC2) and used the same method to confirm that a subset of OPCs expressed *Gpr17* in both the gray and white matter in vivo (58.77% ± 3.19%; Fig. 6C,D). Since we did not detect any sex differences in our single-cell sequencing, samples from both males and females were combined for these RNAscope experiments. We were therefore able to detect and validate, using the novel selected cluster markers and canonical OPC genes, each cluster of OPCs in vivo. In these analyses, we observed both clusterin-expressing OPCs and GPR17-expressing OPCs in each brain region we investigated (cortex, hippocampus, corpus callosum, and cerebellum). This indicates that different brain regions do not seem to only contain one subtype of OPC, nor do these clusters seem to be gray or white matter specific. Future, more in depth, studies should investigate whether proportions of these two OPC subtypes vary between brain regions.

### *Clusterin* and *Gpr17* are exclusively expressed in OPC1 and OPC2 subsets respectively

**Figure 7:**
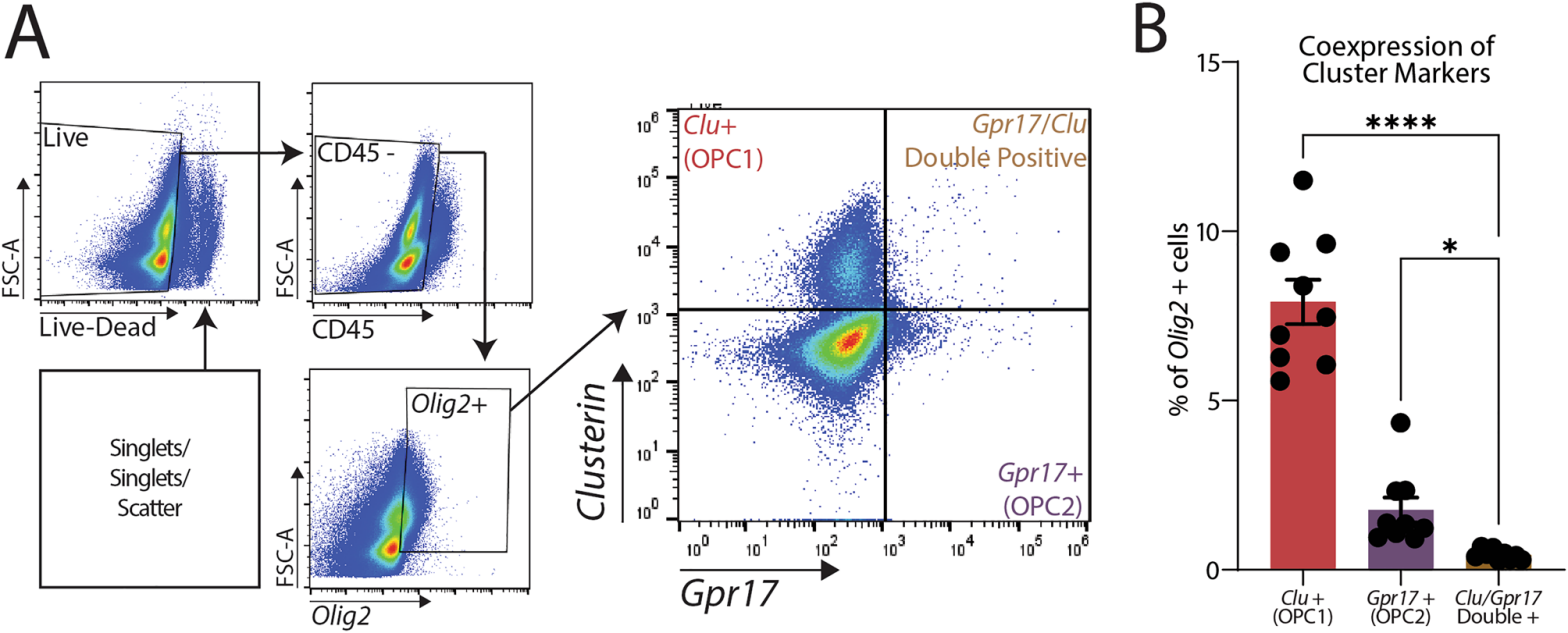
OPC clusters do not overlap in vivo. (**A**) Representative PrimeFlow gating of brain cells stained for CD45 protein and *Clu*, *Gpr17*, *Pdgfra*, and *Olig2* RNA (115,783 events plotted in *Clusterin* vs. *Gpr17* plot). (**B**) Quantification of live/CD45−/Olig2+ cells that express *Clu* alone, *Gpr17* alone, or both *Clusterin* and *Gpr17*. N = 2 individual experiments, n = 9 biological samples (9 males). Analyzed using one-way repeated measures ANOVA with a Tukey’s pos-hoc test (F(2,8) = 90.85, p < 0.0001). *p < 0.05, ****p < 0.0001. Error bars represent SEM. All gates for RNA probes were drawn using fluorescence minus one (FMO) stains.

While RNAscope of canonical OPC markers within each cluster have demonstrated that these clusters of OPCs are expressed within the brain and belong to the oligolineage, neither of these techniques have demonstrated that these markers characterize clusters that are unique from one another. Using PrimeFlow, a technique that allows for the combination of cellular-resolution RNA detection with the multiplexing capabilities of flow cytometry, we demonstrate that a subset of *Olig2* expressing cells express *Clusterin* (OPC1), and a mutually exclusive population expresses *Gpr17* (OPC2), with very few OPCs expressing detectable levels of both cluster markers (Fig. 7A,B; Supplemental Fig. 1). While this does not rule out the possibility that an individual OPC might express genes enriched in different clusters at different times, it does demonstrate that, at any given point, genetic markers of these two clusters of OPCs largely do not overlap.

### Parenchymal fibroblasts

**Figure 8:**
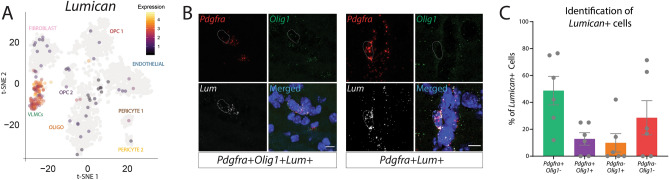
*Lumican*+ fibroblast-like cells are found in the brain parenchyma. (**A**) Expression of *Lumican*, a marker of the VLMC cluster overlaid on the tSNE map. (**B**) Representative RNAscope images of an OPC (*Pdgfra*+ *Olig1*+) and a fibroblast-like cell *(Pdgfra*+ *Olig1*−) expressing *Lumican* in the brain parenchyma. White dotted line indicates the nuclear outline. (**C**) Quantification of the percentage of *Lumican*+ cells in each mouse that also expressed *Pdgfra*, *Olig1*, both, or neither. N = 3 individual experiments, n = 6 biological samples (3 males and 3 females).

While our dataset contains the typical brain-resident PDGFRα-expressing cells (OPCs and endothelial cells) as well as cell types known to arise from PDGFRα-expressing progenitors (oligodendrocytes and pericytes), it also contained a cluster of fibroblasts, likely arising from the meninges as these cells expressed markers recently attributed to the dural meninges, as well as a cluster of cells transcriptionally similar to those previously defined as VLMCs^29,30,33,41,50^. VLMCs are actively expressing significant amounts PDGFRα, but do not express other markers of OPCs or endothelial cells, the most significant expressers of PDGFRα in the CNS (Fig. 3B). Based on this expression and the transcriptional similarity of this VLMC cluster to a previously described cluster of “fibroblast-like” CNS-resident cells, we postulated that these cells may represent parenchymal fibroblasts (Supplemental Table 1)^51^. To validate that this cluster of cells resides in the parenchyma and not the meninges, we used RNAscope to stain the brain for *Lumican*, one of the top genes upregulated in the VLMC cluster (Fig. 8A), as well as for *Pdgfra* and the oligolineage marker *Olig1*. Indeed, we observed cells within the brain parenchyma that expressed *Lumican* and *Pdgfra* but lacked expression of *Olig1* and are therefore not OPCs (Fig. 8B). While we observed some OPCs (*PDGFRα*+ *Olig1*+) that expressed *Lumican*, approximately half (48.73% $\pm$ 10.58%) of all *Lumican*+ cells expressed *Pdgfra*, but no *Olig1* (Fig. 8C). In sum, we have demonstrated that a population of VLMCs, as identified by their co-expression of *Lumican* and *Pdgfra* but lack of *Olig1* expression, resides within the brain parenchyma and likely represents a novel population of brain-resident fibroblasts.

## Discussion

With the development of novel tools that allow for the analysis of tissue at single-cell resolution, interest has surged in outlining how cell types that express the same canonical cell type markers may represent more diverse subpopulations than previously thought^30,52–55^. Here, we demonstrate that OPCs from the adult brain cluster into two distinct subpopulations, characterized by unique transcriptional signatures and Gene Ontology profiles. Furthermore, we demonstrate that a population of fibroblast-like cells reside within the parenchyma.

A small number of studies have investigated the transcriptional profiles of OPCs present during development and have described relatively little transcriptional diversity. Marques et al. transcriptionally profiled oligolineage cells from both juvenile and adult brains and, while mature oligodendrocytes clustered into seven subpopulations, their data indicated one population of progenitor cells and one population of committed oligodendrocyte progenitors (COPs)^30^. While more in-depth lineage-tracing studies are needed to meaningfully determine if OPC1 and OPC2 represent two populations of oligolineage at different stages of differentiation, or if they are two distinct progenitor populations, we think that it is unlikely that our OPC clusters represent a population of OPCs and a population of COPs. For example, Marques et al. note that their population of COPs do not express the OPC gene *Pdgfra*, while we see this transcript in both of our OPC populations. While *Gpr17* was enriched in their population of COPs as well as our OPC2 population, they observed a decrease in cell cycle genes as well as an increase in genes involved in migration (such as *Tns3* and *Fyn*) in this population, while we observed a subpopulation of OPC2 cells demonstrating an enrichment of cell cycle genes (Fig. 4D) and no enrichment in migration genes (Supplemental Table 2). Lastly, we found clusterin significantly distinguished our 2 OPC populations, while it was not differently expressed between their OPCs and COPs. Overall, our data demonstrate the existence of two distinct OPC populations in the adult brain, and further work will be necessary to discover the transition from one population of developmental OPCs to two populations of adult OPCs.

A more recent study from the same group characterized the transcriptional profiles of OPCs from E13.5, E17.5, and P7 mice and found three clusters of OPCs that shared similar transcriptional signatures, but largely clustered by the age of the cells, with one cluster of cycling OPCs^33^. From these data, they concluded that, during development, the three known waves of developmental OPCs converge into a transcriptionally homogenous group of OPCs by P7. Importantly, this sequencing dataset only profiles prenatal and early postnatal OPCs, a time window in which OPCs are preparing to generate a large population of mature oligodendrocytes to support the developmental myelination that occurs during early postnatal time points^28^. Therefore, it is likely that OPCs during this early stage of development may represent a relatively homogenous population of progenitors destined to give rise to myelinating glia^28^. However, following developmental myelination, oligodendrocytes represent a relatively stable population that require minimal replacement, yet OPCs continue to represent approximately 5% of cells in the adult brain and tile every brain region^2,56^. It is therefore reasonable to hypothesize that as the CNS matures, and no longer requires the production of large numbers of mature oligodendrocytes, OPCs may develop diverse transcriptional repertoires, as demonstrated here, to perform alternative functions throughout adulthood.

This idea of functional diversity of OPCs is supported by recent data from Spitzer et al. demonstrating that OPCs throughout the brain express a diverse array of electrophysiological properties and ion channels which become more diverse with age^13^. Additionally, data obtained from zebrafish has demonstrated that OPCs can be categorized into two functionally distinct subpopulations that demonstrate different calcium dynamics^57^. Interestingly, one population of OPCs was found to rarely differentiate in vivo, although these cells maintained their differentiation capacity, indicating that the main function of this population of OPCs is likely something other than serving as a progenitor pool for mature oligodendrocytes^57^.

Recent work using human cells have also indicated transcriptionally diverse populations of OPCs in the adult brain. Recent sequencing data from human Alzheimer’s disease patients and healthy controls demonstrated that healthy controls have three subpopulations of OPCs, and that one of these populations expressed high levels of *Clusterin*, one of the genes we identified as significantly upregulated in OPC1^58^. Additionally, single-cell sequencing data from human patients at fetal, adolescent, and adult timepoints reveal multiple transcriptionally distinct populations of oligo-lineage cells that largely clustered based on the age of the patient^59^.

There may be numerous reasons for the differences observed between our dataset and other sequencing datasets demonstrating a homogenous population of OPCs. This may include factors such as the age of mice at the time of cell collection, the genetic reporter used to enrich for oligolineage cells, and the different proportions of OPCs and mature oligodendrocytes included in the analysis. However, we believe that the addition of our study detailing the transcriptional profile of adult OPCs to previously published datasets will provide the field with a better understanding of how OPCs might change as the brain matures.

Within our single-cell sequencing dataset we also identified a population of cells that reside in the parenchyma and express fibroblast markers. This data supports a recently published report of “fibroblast-like” cells within the brain^51^. While the field has relatively little understanding of either the homeostatic or pathogenic role of these cells, multiple datasets documenting their residence within the parenchyma bolsters support for further study of these cells.

Many of the differentially expressed genes and related biological processes found in each OPC cluster complement emerging literature that indicates non-canonical roles for OPCs during homeostasis, and a more active role of this cell type in multiple diseases. For example, OPC1 expresses high levels of *Clusterin*, a gene known to be upregulated in both Alzheimer’s disease and multiple sclerosis (MS)^60,61^. Interestingly, prior to our data, clusterin was thought to be expressed only by astrocytes and endothelial cells in the brain and, as such, the effect of clusterin on OPC function has not yet been investigated^62,63^. OPCs have recently been shown to potentially play an active role in the pathology of MS and have been implicated in the progression of Alzheimer’s disease^17,20,64^. Given these associations with Alzheimer’s disease and MS, and the known role of clusterin in multiple CNS pathologies, interrogating effects of clusterin on OPC function is useful for our understanding of how these cells may be affecting disease progression. For example, clusterin has been shown to aid in debris clearance and increase phagocytosis in multiple cell types. Since OPCs have been shown to be able to engulf myelin debris, it is possible that clusterin may direct OPCs towards a more phagocytic phenotype^65^. However clusterin may be affecting OPCs, interrogating the functions of this cluster of OPCs will be important in revealing novel ways OPCs help maintain homeostasis and how they subsequently may be playing an active role in contributing to or protecting against CNS pathology^64,66–68^.

OPC2 shows significant upregulation of the G-protein coupled receptor *Gpr17*. Importantly, *Gpr17* is the only documented marker of molecular diversity in OPCs described to date and is only found in one cluster of OPCs in our dataset^45,46^. Previous work has shown that GPR17 functions to prevent OPCs from maturing into oligodendrocytes^69^. GPR17+ OPCs have been shown to react strongly to any type of central nervous system (CNS) insult, as these cells have been found to surround lesions or injuries and begin to proliferate in response to damage^46,69–71^. This behavior of GPR17+ OPCs in response to CNS damage has led to the hypothesis that GPR17+ OPCs are maintained in the adult brain in an immature state as a population of cells prepared to sense and quickly respond to damage^72^.

OPC2 also showed unique upregulation of genes related to neuronal differentiation and synapse organization. These processes are particularly intriguing given that OPCs are the only known glial cell to form canonical synapses with neurons and have recently been shown to be critical in regulating circuit formation during development^12,73^. Birey et al. demonstrated that ablating OPCs significantly altered neuronal function and resulted in depressive and anxiety-like behavior^14^. It remains to be seen if this effect of OPC loss on neuronal function is mediated through another cell type. Yet those studies, coupled with the sequencing data described here, makes investigation of this subpopulation of OPCs crucial in understanding how OPCs are directly influencing neuronal health, circuit functioning and formation, and overall behavioral outcomes.

Interestingly, our RNAscope data demonstrates that brain regions contain cells from both the OPC1 and OPC2 cluster. Furthermore, neither of these clusters were restricted to only gray or white matter regions. While this data indicates that that these clusters of OPCs are not specific to individual brain regions, it does not rule out more subtle spatial differences in these two OPC clusters. For example, one cluster of OPCs may more commonly be found near neuronal cell bodies or blood vessels. Future experiments should utilize new methods, such as spatial transcriptomics, that will allow for the staining of many genes of interest in addition to providing spatial information, to further describe potential spatial differences in these cell types.

Many of the differentially expressed genes and related biological processes found in each OPC cluster complement emerging literature that indicates non-canonical roles for OPCs during homeostasis, and a more active role of this cell type in multiple diseases. While here we describe the transcriptional profile of OPCs during homeostasis, it is important to note that understanding the role of OPCs in the healthy brain will provide a necessary foundation for examining any protective or detrimental novel functions in disease pathology. We believe that the work presented here provides a critical foundation and basis for the investigation of non-canonical roles of OPCs. This dataset will not only assist the field in discovering novel roles for OPCs in both health and disease, but can also offer potential mechanistic explanations for intriguing phenotypes observed in OPC deletion paradigms^14–17^.

## Supplementary Information

Supplementary Figure 1.

Supplementary Table 1.

Supplementary Table 2.

Supplementary Table 3.

## Data Availability

All analyzed single-cell sequencing data has been uploaded in a searchable database located at http://165.22.7.10:3838/seurat_viewer/seurat_viewer_4.Rmd. The sequencing data has been deposited in the National Center for Biotechnology Information (NCBI) Gene Expression Omnibus (GSE147075). All other datasets used within this publication are available from the corresponding authors upon reasonable request.
