# Supplementary material for: Evidence for oligodendrocyte progenitor cell heterogeneity in the adult mouse brain
Source: Sci Rep. 2022 Jul 28;12:12921. doi: 10.1038/s41598-022-17081-7 (PMC9334628; doi:10.1038/s41598-022-17081-7)
Supplement: Supplementary file 1 — Supplementary Figure 1. [file 41598_2022_17081_MOESM1_ESM.pdf]

***Evidence for oligodendrocyte progenitor cell heterogeneity in the adult mouse brain*.**

Authors: Rebecca M. Beiter[1,2], Courtney Rivet-Noor[1,2], Andrea R. Merchak[1,2], Robin Bai[1], David M. Johanson[1], Erica Slogar[1], Katia Sol-Church[3], Christopher C. Overall[1], and Alban Gaultier[1,2#]

[1]Center for Brain Immunology and Glia, Department of Neuroscience, [2]Graduate Program in Neuroscience, [3]Genome Analysis and Technology Core, University of Virginia School of Medicine, Charlottesville, VA 22908, USA,

[#]Corresponding author. Email: ag7h@virginia.edu

Supplemental Figure 1

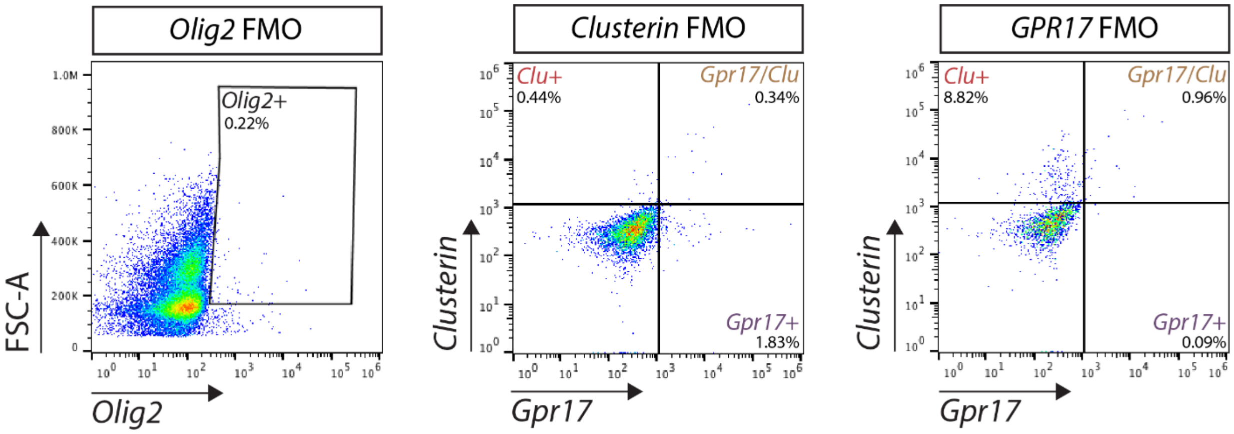

Supplemental Figure 1: Fluorescence minus one (FMO) samples used to draw RNA flow cytometry gates in Figure 7. Flow plots from FMO controls for all RNA probes used in Figure 7. These controls were used to accurately draw the gates on the flow plots to ensure that false positives were not contained within our gates. Percentages included on the plots are the percent of cells that fall within that gate. The number of events plotted in each flow plot are: *Olig2* FMO – 34, 867 events; *Clusterin* FMO – 5,032 events; GPR17 FMO – 2,178 events.
